# RNA-sequencing studies suggest that microRNAs and alternative splicing of pre-mRNAs modulate immune and inflammatory responses in Holstein cattle infected with *Mycobacterium avium* subsp. *paratuberculosis*

**DOI:** 10.3389/fimmu.2025.1597736

**Published:** 2025-06-25

**Authors:** Gerard Badia-Bringué, Marta Alonso-Hearn

**Affiliations:** Department of Animal Health, NEIKER-Basque Institute of Agricultural Research and Development, Basque Research and Technology Alliance (BRTA), Derio, Bizkaia, Spain

**Keywords:** *paratuberculosis*, miRNAs, alternative splicing, gene regulation, biomarkers

## Abstract

RNA-Sequencing (RNA-Seq) studies using bovine samples from *Mycobacterium avium* subsp. *paratuberculosis* (MAP)-infected animals have identified a range of differentially expressed mRNAs with potential as biomarkers for MAP infection. As bioinformatic tools continue to develop, microRNAs (miRNAs) and alternative splicing (AS) have emerged as important contributors to host responses during infections. Although RNA-Seq enables AS analysis, most transcriptomic studies still emphasize gene-level expression due to the complexity of AS workflows, the need for deeper sequencing, and incomplete transcript annotations, even in humans. Nevertheless, interest in AS is growing, driven by its recognized role in tissue-specific functions and disease mechanisms. Recent findings have revealed distinct miRNAs and AS profiles in MAP-infected cattle compared to uninfected cows, indicating that post-transcriptional regulatory mechanisms are altered during MAP infection. In this comprehensive review, we examine critical aspects of post-transcriptional regulation in the context of MAP infection. We focus on recent studies investigating miRNAs and AS profiles, highlighting their roles in modulating immune responses and their potential as novel diagnostic biomarkers. Notably, overlapping mechanisms involving miRNAs and AS have been identified in paratuberculosis and in several human diseases, suggesting conserved pathways of host-pathogen interaction and immune regulation.

## 1 Bovine paratuberculosis

PTB is an infectious chronic disease caused by *Mycobacterium avium* subsp. *paratuberculosis* (MAP), which affects ruminants worldwide. Due to its socio-economic importance, notification to the World Organization for Animal Health (WOAH) is obligatory (1). PTB is distributed worldwide, with herd-level prevalences higher than 50% in the USA and Europe (2). Clinical signs of the disease include diarrhea, weight loss, decreased milk production, and premature culling (1). Economic losses due to PTB can be directly caused by the clinical signs of the disease, such as animal death or decrease in milk production, or by indirect consequences, such as the cost of the diagnostic testing of all the animals in the herd or the premature culling of animals. In a recent study, Rasmussen et al. estimated the 10-year average annual loss per cow due to PTB at 53.30 US$ in the USA, 34.40 US$ in Great Britain, 33.45 US$ in Italy, 48.91 US$ in Canada, 31.25 US$ in Australia, 45.99 US$ in Spain, 21.72 US$ in Belgium, 8.31US$ in Brazil, and 81.53US$ in Japan (3). These losses represent between 0.72% and 1.41% of the annual milk revenue. Estimates of global annual losses due to PTB currently can reach up to 4–5 billion US$ (4).

MAP also poses a potential risk to human health. There are similarities between the histopathological lesions and clinical signs that appear in PTB-infected cattle and human patients with gastrointestinal inflammatory diseases such as Crohn’s disease (CD) and inflammatory bowel disease (IBD) (5–7). Moreover, MAP has been identified in blood and tissue samples from patients with CD (8–10), and in the milk of pregnant women with CD (11). Additionally, anti-mycobacterial antibiotics can induce remission of pediatric CD (12). However, a causal association between MAP and CD has not been demonstrated (13). Recent studies have also shown an association between MAP and other human diseases such as type-1 diabetes (14), sarcoidosis (15), Alzheimer’s disease (16), rheumatoid arthritis (17), Hashimoto’s thyroiditis (18), Blau’s syndrome (19), multiple sclerosis (20) and cancer (21, 22). Colorectal cancer can appear as the result of complications of the two forms of idiopathic IBD: CD and ulcerous colitis (23).

## 2 RNA-Sequencing: a powerful tool to discover novel biomarkers

PTB control measures include the culling of MAP-infected animals and the enhancement of farm biosecurity measures. The current diagnostics tools for the direct detection of MAP are fecal real-time quantitative polymerase chain reaction (qPCR) and bacteriological culture, with the latter being considered the gold standard. However, both show limitations in detecting subclinical MAP-infected cattle with low bacterial load in feces and gut tissues. The lack of sensitivity of current diagnostic tests poses a limitation mainly for the identification of cattle with subclinical infection, which requires the performance of periodic tests and increases costs. The detection of subclinical infections remains a challenge, and novel tools are needed to detect MAP-infected animals at the subclinical stages of the disease. RNA sequencing (RNA-Seq) is a high-throughput sequencing technique that provides data on the transcriptome of a cell or tissue. RNA-Seq can provide biological information on how an animal responds to an infection and is a source of novel biomarkers. RNA-Seq studies have used different samples from MAP-infected cattle, including those from the ileocecal valve (ICV) (24–26), salivary glands (27), whole blood (26, 28), Peyer’s patches (29), and jejunum and ileum (30). Other RNA-Seq studies used MAP-infected macrophages (31–36) and human monocytic cell lines differentiated to macrophages (37, 38). The mRNA of the ABCA13 transporter — previously identified in subclinical MAP‑infected cattle — has been validated as a serum biomarker for detecting Holstein cows with focal PTB‑associated lesions (26, 39). A summary of RNA-Seq studies using MAP-infected macrophages and bovine samples from MAP-infected animals is presented in **Table 1**.

**Table 1:** RNA-Seq studies that identified biomarkers associated with MAP infection.

Sample type	Group	N° of animals	Reference
Ileocecal valve	Control	5	(24)
	Subclinical infection	5	
	Clinical infection	5	
	Infected	5	(25)
	Uninfected	5	
	Without lesions	3	(26)
	With focal lesions	5	
	With diffuse lesions	5	
Bovine macrophages	Uninfected	7	(31)
	2h p.i.	7	
	6h p.i.	7	
	Uninfected	68	(32)
	2h p.i.	68	
	6h p.i.	68	
	24h p.i.	68	
	Uninfected	3	(33)
	6h p.i.	3	
	Uninfected	6	(34)
	Infected	6	
	Uninfected	6	(36)
	6h p.i.	6	
Salivary glands	Infected at 1, 4, 8 and 24h p.i.	18	(27)
	Uninfected at 1, 4, 8 and 24h	6	
Whole blood	Infected	5	(28)
	Uninfected	5	
	Without lesions	3	(26)
	With focal lesions	5	
	With diffuse lesions	5	
Peyer´s patches	Infected	10	(29)
	Uninfected	5	
Jejunum and ileum	Infected	4	(30)
	Uninfected	3	
Human macrophages	3h p.i.	THP-1 cells*	(37)
	Uninfected	THP-1 cells*	
	3h p.i.	2	(38)
	Uninfected	2	

*Monocyte cell line isolated from peripheral blood of an acute monocytic leukemia patient.

Other potential biomarkers that can be used for MAP diagnosis include non-coding RNAs (ncRNAs) like microRNAs (miRNAs). miRNAs are post-transcriptional regulators of gene expression through their binding to their target mRNAs. Biomarkers associated with specific stages of MAP infection can also include transcript isoforms, caused by alternative splicing (AS). AS of pre-mRNA consists of the removal of introns and the assembly of exons contained in eukaryotic genes. AS events can influence transcript stability or structure with important physiological consequences. While RNA-Seq has enabled AS analysis, most transcriptomic studies still focus on gene-level expression rather than transcript isoform diversity. AS analysis requires more complex computational pipelines, greater sequencing depth, and careful interpretation. There is also the challenge of incomplete annotations of all transcript isoforms, even in well-studied organisms like humans. However, this area is gaining more attention, especially with the growing understanding of how crucial AS is to tissue-specific functions and disease mechanisms.

## 3 MiRNAs

MiRNAs are highly conserved small ncRNAs (18 to 25 nucleotides) that regulate the expression of their target mRNAs. The first identified miRNA was detected in *Caenorhabditis elegans* by Lee et al. and was named lin-4 as it is a regulator of the LIN-14 protein (40). Years later, miRNAs were found in abundance in different invertebrate and vertebrate species with a high degree of conservation, which suggested that they were associated with important regulatory processes common between species.

### 3.1 Biogenesis of miRNAs

**Figure 1:**
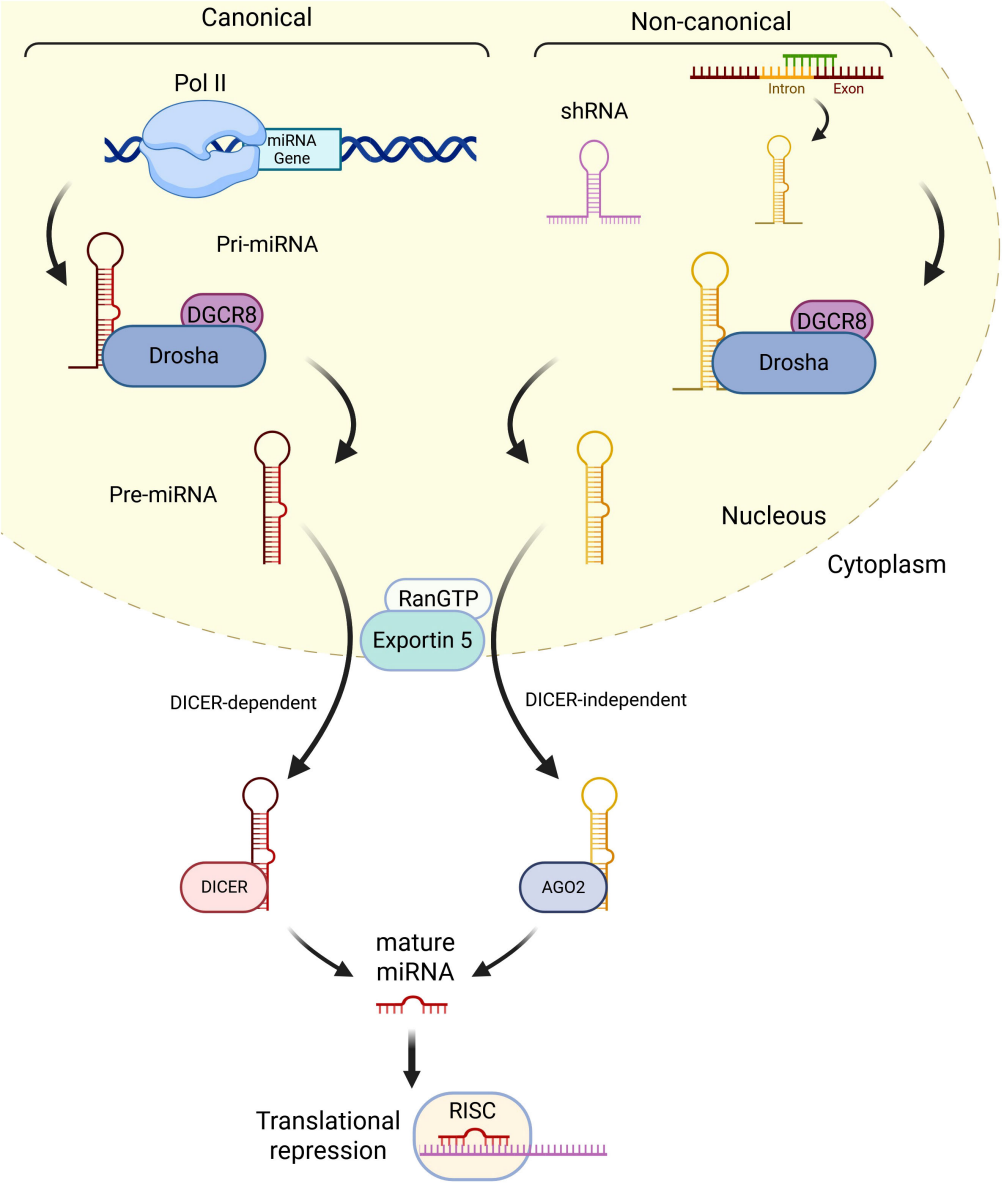
Summary of miRNA biogenesis. In the canonical pathway, miRNAs are usually transcribed by RNA polymerase II and generate pri-miRNAs, which are cleaved by DGCR8 and DROSHA into a stem-loop structure called pre-miRNA. RanGTP/Exportin 5 complex translocates the pre-miRNA to the cytoplasm, where it is processed by DICER into a miRNA duplex containing the mature miRNA and the passenger miRNA. After the duplex is undone, the mature miRNA is incorporated into the RISC complex and can induce gene silencing and/or mRNA degradation. In the non-canonical pathway, shRNAs are initially cleaved by the microprocessor complex and exported to the cytoplasm via RanGTP/Exportin5. They are further processed via AGO2-dependent, but DICER-independent, cleavage. All pathways ultimately lead to a functional miRISC complex. In most cases, miRISC binds to target mRNAs to induce translational inhibition (Created in Biorender.com).

MiRNA biogenesis is classified into the canonical and non-canonical pathways (41) (**Figure 1**). In the canonical pathway, miRNA synthesis starts with a large primary transcript (pri-miRNA), an RNA stem-loop with a cap at 5’, and a polyadenine at 3’, usually transcribed by the RNA polymerase II (42). The 5’ cap and 3’ polyadenine of the pri-miRNA are cleaved in the cellular nucleus by a microprocessor complex formed by the type-III RNase Drosha and a double-stranded RNA binding protein, *DiGeorge syndrome critical region 8* (*DCGR8*) in mammals, to produce the pre-miRNA, which only contains the stem-loop structure (43). The pre-miRNA is translocated to the cytoplasm by the Ran/GTP/Exportin 5 complex, where it is processed by Dicer, another type-III RNase, which breaks the stem-loop and forms a miRNA-miRNA duplex. One of the strands is the mature miRNA, while the other is the “passenger” strand (44). The non-canonical pathway usually starts with an ncRNA, such as an intron or a short hairpin RNA (shRNA). The shRNA is initially cleaved by the microprocessor complex and exported to the cytoplasm via Ran/GTP/exportin5. The pre-miRNAs are further cleaved through the Argonaute-2 (AGO2), but Dicer-independent, cleavage (45). Mirtrons, which are produced from the introns of mRNAs during splicing, and 7-methylguanosine (m^7^G)-capped pre-miRNAs are dependent on Dicer to complete their cytoplasmic maturation, but they differ in their nucleocytoplasmic shuttling. Mirtrons are exported via Exportin5/Ran/GTP, while m^7^G-pre-miRNA are exported via Exportin1. The mature miRNA generated from the canonical or non-canonical pathways is integrated into the RNA-induced silencing complex (RISC), which guides the miRNA to its target mRNA. The binding of miRNAs to their target mRNAs induces gene silencing and/or mRNA degradation. The binding of miRNAs to the 5’-untranslated region (5’UTR) and other DNA elements could enhance translation and transcription (46). miRNAs tend to associate with proteins and/or travel in serum and plasma encapsulated within vesicles, which makes them more resistant to degradation than other RNAs and allows them to be measured with high sensitivity (47).

### 3.2 MiRNA profiling in MAP infection

The first study analyzing miRNA expression in MAP-infected animals did not show differential miRNA patterns between interferon-γ (IFN-γ) responders and unchallenged controls, probably due to limitations of the available miRNA annotation (48). However, the authors did identify differential expression of miR-205, miR-10b, miR-92b, miR-432, miR-27a, miR-127, miR-126, and miR-143 between controls at time point zero and controls at 6 months post-infection. These miRNAs are associated with proliferation and development roles in different mammal species, which suggests that miRNA expression also changes during development processes. Shaughnessy et al. did not detect differentially expressed (DE) miRNAs in serum at either the early (6 months) or late (43, 46, and 49 months) intervals between seropositive and seronegative animals. However, some miRNAs, such as bta-miR-92b, bta-miR-29a, bta-miR-205, and bta-miR-345-3p, were DE in seropositive animals at time point 0 and time points 6, 43, 46, and 49 months. These miRNAs are associated with immune functions, suggesting that they could be associated with immune maturation.

A summary of the miRNAs with differential expression between MAP-infected and uninfected cattle is shown in **Table 2**. Wang et al. identified DE miRNAs in monocyte-derived macrophages (MDMs) infected with MAP 6 hours after infection (49). These DE miRNAs were associated with modulation of the MAPK pathway (miR-12023 and miR-1343-3p), the NF-kβ pathway (miR-214, bta-miR-133a, and bta-miR-1246), and apoptosis (bta-miR-1246, bta-miR-1306, bta-miR-454, and bta-miR-150. Using BoMac cells and MDMs, Wright et al. showed that MAP can modulate miR-19a, miR-129, miR-24, and miR-24-3p expression to induce macrophage polarization (50). MiR-19a and miR-129 are associated with an M1 macrophage phenotype and regulate lipid efflux and inflammation. MiR-24-3p, however, inhibits phagocytosis and promotes the M2 macrophage phenotype. These findings suggest that MAP can interfere with inflammation and macrophage function by altering miRNA expression. Additional studies in MAP-infected bovine macrophages found that bta-miR-133b, bta-miR-92a, and bta-miR-184 were downregulated upon MAP infection (51). Target gene function analysis suggested that miR-92a was involved in interleukin signaling, hence regulating the immune response against MAP.

**Table 2:** MiRNAs that are dysregulated upon MAP infection.

Sample type	miRNAs	Up/downregulation	Role of the miRNAs	Reference
Macrophages	bta-miR-92b, bta-miR-29d-5p, bta-miR-454, bta-miR-1306, bta-miR-1343-3p, bta-miR-2443, and bta-miR-12023	Downregulated	MAPK, NF-kβ, and apoptosis	(49)
	bta-miR-132, PC-3p-7159_279, bta-miR-1246, bta-miR-2484, bta-miR-150, bta-miR-451, bta-miR-214, bta-miR-677, bta-miR-380-3p, bta-miR-122, bta-miR-212, bta-miR-1434-5p, bta-miR-369-3p, and bta-miR-133a	Upregulated		
	miR-129-5p	Downregulated	Macrophage polarization, lipid efflux, and inflammation, phagocytosis	(50)
	miR-129-5p, miR-19b-3p, and miR-24-3p	Upregulated		
	bta-miR-133b, bta-miR-92a, and bta-miR-184	Upregulated	Interleukin signaling	(51)
Whole blood	bta-mir-19b, bta-mir-19b-2, bta-mir-1271, bta-mir-100, bta-mir-301a, bta-mir-32, 14_7917(Novel), and 27_25982(Novel)	Downregulated	Phagocytosis, macrophage function, and cytokine production	(52)
	bta-mir-6517, bta-mir-7857, bta-mir-24-1, bta-mir-24-2, and bta-mir-378c	Upregulated		
Ileal segments	bta-miR-105a, bta-miR-433, bta-miR-2400, bta-miR-137, bta-miR-424–3p, bta-miR-138, and bta-novel-53(Novel)	Downregulated	TLR signaling, lymphocyte activation, inflammatory response, and phagocytosis	(25)
	bta-miR-146b, bta-miR-196b, bta-miR-2483–5p, bta-miR-133b, bta-miR-1247-5p, bta-miR-184, and bta-miR-202	Upregulated		
Feces	miR-501-5p, miR-658	Downregulated	ND	(53)
	miR-92a-3p	Upregulated		
Sera	bta-miR-147, bta-miR-196a, bta-miR-346, bta-miR-655, and bta-miR-2426	Downregulated	Ras and Wnt pathways	(54)
	bta-miR-363, bta-miR-374b, bta-miR-2887,	Upregulated		
	and miR-92a-3p			
Whole blood diffuse lesions *vs.* no lesions	bta-miR-144, bta-miR-19a, and bta-miR-101	Downregulated	Not identified	(56)
	bta-miR-2425-3p, and bta-miR-139	Upregulated		
Whole blood diffuse *vs.* focal lesions	bta-miR-144, bta-miR-19a, and bta-miR-32	Downregulated	Not identified	
Ileocecal valve focal lesions *vs.* no lesions	bta-miR-23b-3p and bta-miR-23a	Downregulated	RNA polymerase	
	bta-miR-2478 and bta-miR-150	Upregulated		
Ileocecal valve diffuse lesions *vs.* no lesions	bta-miR-135b and bta-miR-433	Downregulated	MAPK signaling	
Ileocecal valve diffuse *vs.* focal lesions	bta-miR-204, bta-miR-99a-5p, bta-miR-10174-3p, bta-miR-154c, bta-miR-433, bta-miR-23b-3p, bta-let-7e, bta-miR-214, bta-miR-23a, bta-miR-382, bta-miR-145, bta-let-7c, bta-miR-30a-5p, bta-miR-27b, and bta-miR-411a	Downregulated	MAPK, P53, lysosome, PI3K/Akt, JAK-STAT, and regulation of immune system	
	bta-miR-146a, bta-miR-146b, and bta-miR-10167-3p	Upregulated		

An analysis of whole-blood samples collected from MAP-infected cows and uninfected cows from a MAP-free herd based on ELISA results and absence of clinical cases revealed differential expression of miRNAs with roles in the regulation of the immune response and inflammation upon bacterial or viral infection (52). In the infected cows, downregulation of bta-miR-100 and bta-miR-301a and upregulation of bta-miR-32 were observed. The targets of these miRNAs associated with phagocytosis, macrophage function and cytokine production were the *Tweety Family Member 3* **(** *TTYH3)*, *HIC ZBTB Transcriptional Repressor 1* (*HIC1)*, *Inosine Monophosphate Dehydrogenase 1* (*IMPDH1)*, *ARF Like GTPase 2* (*ARL2)*, *Adaptor Related Protein Complex 2 Subunit Alpha 1* (*AP2A1)*, and *Zinc Finger and BTB Domain Containing 4* (*ZBTB4)*. Other differentially expressed miRNAs were bta-mir-19b, bta-mir-19b-2, bta-mir-1271, and a novel miRNA (Novel:14_7917). The authors hypothesize that the identified miRNAs may be involved in regulating early immune responses.

MAP infection can also result in miRNA upregulation in the ileal segments of newborn calves, such as miR-146b, which inhibits proteins in the TLR signaling pathway such as interleukin-1 receptor-associated kinase 1 (IRAK1), interleukin-1 receptor-associated kinase 2 (IRAK2), and tumor necrosis associated factor 6 (TRAF6) (25). Therefore, MAP could be interfering with TLR signaling and downstream inflammatory responses. This study also found an upregulation of bta-miR-202, bta-miR-184, bta-miR-196b, and bta-miR-1247, associated with lymphocyte activation, muscle and epithelium development, inflammatory response, the proliferation of endothelial cells, phagocytosis, and epigenetic regulation. Downregulation of bta-miR-133b, bta-miR-433, bta-miR-105a, and bta-miR-137, associated with lymphocyte activation, the proliferation of endothelial cells, muscle and epithelium development, and vesicle transport, was also detected.

Fecal samples have also been used to identify DE miRNAs between cows with clinical signs of PTB and with a bacteriological culture and ELISA-positive result *vs.* healthy cows (53). The results showed downregulation of miR-501-5p and miR-658, and upregulation of miR-92a-3p. When comparing miRNA-Seq data obtained from serum samples of cows in different stages of PTB based on ELISA and PCR results and clinical signs *vs.* control cows, Choi et al. detected differential expression of the bta-miR-363, bta-miR-196a, bta-miR-147, bta-miR-655, bta-miR-346, and bta-miR-2426, associated with the Ras and Wnt pathways (54). Two miRNAs (bta-miR-374b and bta-miR-2887) were identified in cows with PCR-positive and ELISA-negative results and with key molecules such as interleukin-10 (IL-10) and transforming growth factor beta (TGF-β1).

Previous studies have compared the miRNA profiles of cattle with positive and negative ELISA and fecal PCR results (52–55). In cattle, however, focal lesions in gut tissues can be detected before fecal shedding and ELISA. Therefore, examining the miRNA profiles of MAP-infected animals selected according to the presence of focal histopathological lesions facilitates the identification of miRNAs in animals in the subclinical stage of the infection when the amount of MAP and antibodies cannot be detected with current pre-mortem diagnostic methods. Our research team recently used miRNA-Seq to quantify and compare the expression of miRNAs in ICV and whole-blood samples collected from cows with focal or diffuse PTB-associated lesions in gut tissues *vs.* control cows without lesions (56). To our knowledge, this was the first study that used sRNA-Seq to compare miRNA expression in blood and gut tissues of cattle classified according to histopathological data. None of the identified miRNAs were differentially expressed in both the PB and ICV samples, and, therefore, our results showed PB and ICV-specific miRNAs expression. Specifically, in blood samples, we identified eight miRNAs in the comparison of cows with diffuse lesions *vs.* controls (bta-miR-2425-3p, bta-miR-144, bta-miR-19a, bta-miR-32, bta-miR-139, bta-miR-101, bta-miR-27a-5p, and bta-miR-181a) and three in the comparison of cows with diffuse *vs.* focal lesions (bta-miR-19a, bta-miR-144, and bta-miR-32). Among the eight miRNAs differentially expressed in blood samples from cows with diffuse lesions *vs.* controls, three (bta-miR-19a, bta-miR-144, and bta-miR-32) were also found downregulated in PB samples of cows with diffuse *vs* focal lesions. Interestingly, bta-miR-32 was also downregulated in a previous study where the miRNA profiles of Holstein cattle positive and negative for MAP antibodies were compared (52). In the ICV samples, we identified 4, 5, and 18 miRNAs that were differentially expressed in cows with focal lesions *vs.* controls, diffuse lesions *vs.* controls, and diffuse *vs.* focal lesions, respectively. This suggests that the miRNA expression in ICV changes more as PTB-associated lesions become more severe. In the comparison of cows with focal lesions *vs.* controls, two upregulated (bta-miR-2478 and bta-miR-150) and two downregulated (bta-miR-23a and bta-iR-23b-3p) miRNAs were identified. DE miRNAs in the comparison of cows with diffuse lesions *vs.* controls (bta-miR-433, bta-miR-146a, and bta-miR-99a-5p) were also dysregulated in the comparison of cows with diffuse *vs.* focal lesions. Interestingly, the human ortholog of bta-miR-146a is an inhibitor of the pro-inflammatory immune response (57). We, therefore, speculated that the upregulation of miR-146a would negatively regulate TRAF6 and control the pro-inflammatory immune response (58). Two miRNAs (bta-miR-23a and bta-miR-23b-3p) were downregulated in both comparisons, i.e., in cows with focal lesions *vs.* controls and diffuse *vs.* focal lesions. Some of the miRNAs identified in our study had been previously identified in studies describing miRNA expression in response to mycobacterial infections, including bta-miR-27a-5p, bta-miR-32, bta-miR-23a, bta-miR-135b, bta-miR146a, bta-miR146b, bta-miR-433, bta-let-7e, and bta-let-7c (25, 52, 59, 60).

## 4 Alternative splicing

Splicing is the process by which the pre-mRNA’s introns are removed, and the exons are rearranged.

### 4.1 AS mechanism and types

**Figure 2:**
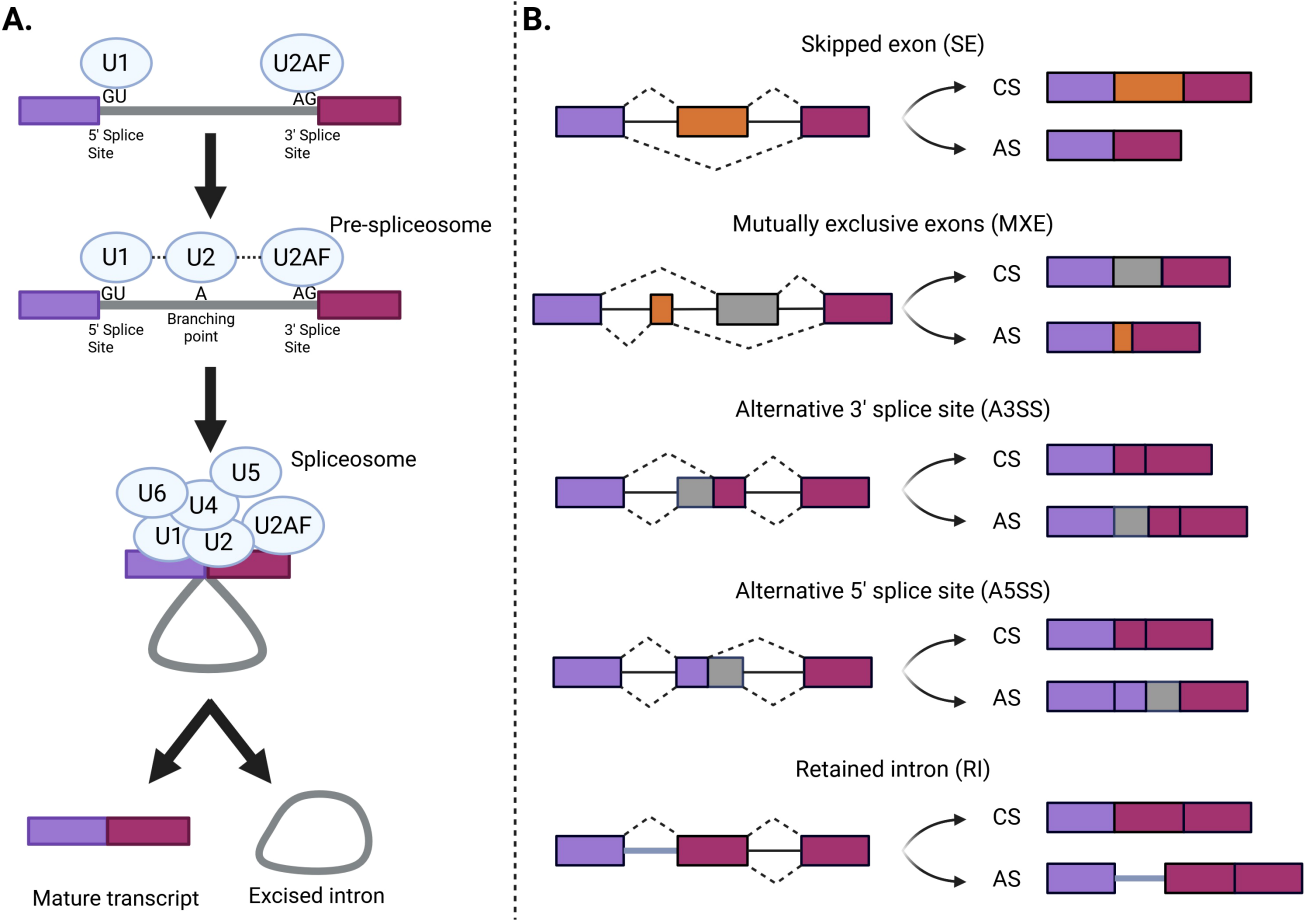
The splicing process and AS events. **(A)** First, a recognition of the 5’ and 3’ splice sites by the U1 and U2 small nuclear ribonucleoprotein complexes (snRNPs) occurs. Then, the U4, U5, and U6 snRNPs are recruited into the active spliceosome. Finally, the functional spliceosome excises the intron and binds the two exons. **(B)** Different types of alternative splicing events. These classes include skipped exon (SE), in which a single exon is included or excluded from the final transcript; mutually exclusive exons (MXE), in which the final transcript only contains one of the two affected exons; alternative 3′ splice site (A3SS), in which the acceptor site (at 3′) is changed; alternative 5′ splice site (A5SS), in which the donor site (at 5′) is changed; and retained intron (RI), in which an intron gets included in the final transcript. Violet and magenta bars represent the constitutive sequence that always forms part of the mature mRNA, while yellow and grey represent the alternative sequences that can be either included or excluded in the mature mRNA. Exons and introns are represented by boxes and solid lines, respectively (Created in Biorender.com). AS, alternative splicing; CS, constitutive splicing.

This process is mediated by the spliceosome, a complex formed by highly dynamic small nuclear ribonucleoproteins (snRNPs) (U1, U2, U4, U5, and U6) and accessory proteins (61–63) (**Figure 2A**). The same mRNA can be spliced in different ways to produce different proteins. Splicing events can be categorized depending on whether they are always (constitutive) or only sometimes (alternative) recognized by the spliceosome and spliced in the mature mRNA. The most frequently used splice sites contain highly conserved sequences easily recognized by the spliceosome and lead to constitutive splicing (CS), whereas less frequently used splice sites lead to AS and are only used when specific splicing regulatory elements are activated. Depending on the splice site locations, AS events can be classified into different classes (**Figure 2B**). AS events can have multiple detrimental consequences in the mature transcript, such as a decrease in mRNA stability, a change in protein function or target, a change in final protein location, and the generation of premature termination codons, which are natural targets of the nonsense-mediated decay pathway and can cause changes in gene expression (64, 65). Changes in the frequency of AS events can be the cause of specific diseases, modify their severity, or be associated with their susceptibility (66).

### 4.2 AS profiling in MAP infection

Genes and pathways associated with AS event changes in MAP-infected cattle have been identified and are summarized in **Table 3**. Using surgically isolated intestinal segments, significant changes in the frequency of two AS events between MAP-infected and non-infected tissues were detected 1 month post-infection (25). There were 16 genes that displayed significantly different AS events between the control and infected compartments of control and infected animals. RT-qPCR was performed to verify the differences between MAP-infected and control tissues in alternative spliced forms of the *adenosine deaminase* (*ADA*) and *monocyte-to-macrophage differentiation-associated* (*MMD*) genes. The identified AS events affected *ADA* and *MMD* mRNAs and appeared more frequently in MAP-infected intestinal segments. The event affecting *ADA* is a retained intron (RI) event, in which the intron 4 was retained significantly more frequently in MAP-infected segments. The event affecting *MMD* consisted of a mutually exclusive exon (MXE) event, in which the alternative first exon was detected more frequently in infected segments. ADA is involved in purine metabolism, and its deficiency results in the accumulation of adenosine in the lysosome, as well as lysosome enlargement, alkalinization, and dysfunction (67). *MMD* is expressed in mature macrophages and participates in macrophage activation through TNF-α and nitric oxide production (68). MAP infection may cause these AS events to disrupt macrophage maturation and lysosome function. The retention of intron 4 in *ADA* could decrease transcript stability, gene expression, and impair macrophage lysosome function. An abnormal first exon of *MMD*, however, could disrupt protein synthesis or function and interfere with macrophage maturation and MAP clearance during the early stage of the infection.

**Table 3:** Pathways affected by AS events associated with MAP infection.

Sample type	Genes that displayed significantly different AS events between control and infected animals	Pathways affected by AS events	Reference
Ileal segments	*ADA* ^1^ and MMD^1^	Macrophage maturation and lysosome function	(25)
Jejunum	*COPG2, KIF2C^2^, EXOC7^2^, RABEPK, DNASE1*, and *EEA1*	vesicle-mediated transport, regulation of acute inflammatory response, and tuberculosis	(69)
Peripheral blood			
Salivary gland			
Peripheral blood	*BOLA, CYTH1, LOC509006, DNM2, CLTA, JSP.1, RAB11FIP5, AP2M1, PRKCI, ARAP1, CD53, CLEC12A, CPNE1, ITGA2B, NCF1, SGSH, SIRPA, SLC11A1, TARM1, TMC6, CLTA, DNM1, DNM2, EPS15L1, FCGR2B, FCHO1, PICALM, NCOA7, DMXL1, SLC11A1, MAN2B1, LAPTM4A, AP3B1, NAGA, CTSW, ACTN1, LOC512286, LOC782367, LOC788175, TGFB2, ITGB2*, and *C8G*	Endocytosis, clathrin-mediated endocytosis, neutrophil degranulation, platelet activation, lysosome, and amoebiasis	(70)
Ileocecal valve ^3^	*BOLA, BOLA-NC1, LOC616942, GRK3, IL2RG, C2, C4A, PSMB10, NR1H3*, and *PSAP, ASAH1*	Endocytosis, antigen processing and presentation, complement activation, humoral immune response mediated by Igs, immune system processes, IgG-mediated immune response, B-cell mediated immunity, and the lysosome	

^1^Validated by RT-qPCR. ^2^Differential AS events in these genes significantly changed protein structure. ^3^Genes displaying changes in both AS and gene expression.

RNA-Seq data from Holstein cows classified as PTB-positive or negative according to ELISA and fecal PCR results allowed the identification of 119 (90 genes), 150 (89 genes), and 68 (45 genes) differential AS events between MAP-infected and healthy Holstein cows in whole-blood, jejunum, and salivary gland samples, respectively (69). Furthermore, 14 genes (*CD3G, SCARB1, MYO5A, RABEPK, KIF2C, EXOC7, COPG2, REPS1, NFYC, SYK, EEA1, CARD9, DNASE1, TAC1*, and *RHBDD3*) were significantly enriched for immune-related pathways, including vesicle-mediated transport, regulation of acute inflammatory response, and tuberculosis. Two of the identified AS events may alter protein structure, with an event in *exocyst complex component 7* (*EXOC7*), which skipped exon 8 and appeared less frequently in MAP-infected animals, and an event in *kinesin family member 2C* (*KIF2C*), which skipped exon 10 and appeared more frequently in MAP-infected animals. EXOC7 plays an important role in vesicular trafficking and the secretory pathway, and it has been reported that a lack of EXOC7 causes a decrease in the number of phagosomes, reducing the efficiency of cytokine transport. *KIF2C* encodes for a protein associated with mitotic chromosome segregation and with immune cell migration and infiltration in humans. Thus, the skipping of exon 10 may affect the immune response against MAP by decreasing immune cell migration.

Our research team has recently used RNA-Seq to identify differential AS profiles between whole blood and ICV samples collected from Holstein cattle with focal and diffuse PTB-associated lesions in gut tissues *v*s cows without lesions in gut tissues (70). The role of AS in the regulation of the immune response was studied while keeping in mind its potential application in the diagnosis of subclinical MAP infection. To the best of our knowledge, our study is the first genome-wide profiling of AS events in whole-blood and ICV samples from Holstein cows with PTB-associated lesions of distinct severity *vs.* uninfected cattle without lesions in gut tissues. In the PB of cows with focal lesions *vs.* controls, differential AS was identified in genes involved in endocytosis (bta041144) including *BOLA*, *LOC509006*, *JSP.1*, *Dynamin-2* (*DNM2*), *CLTA*, *CLTA Adaptor complex AP2*, *Mu subunit* (*AP2M1*), *Cytohesin 1* (*CYTH1*) *RAB11 Family Interacting Protein 5* (*RAB11FIP5*), the apoptosis regulator (*PRKC1*), *ArfGAP With RhoGAP Domain*, and *Ankyrin Repeat And PH Domain 1* (*ARAP1*). CLTA is the main structural component of the lattice-type cytoplasmic face of coated pits and vesicles generated during receptor-mediated endocytosis. DNM2 participates in the release of CLTA-coated vesicles from the plasma membrane, and AP2M1 is required for the activity of a vacuolar ATPase, which pumps protons into endosomes and lysosomes to acidify them. CYTH1 regulates the adhesiveness of integrins at the plasma membrane and is involved in membrane trafficking, and PRKC1 negatively regulates apoptosis. After entry into macrophages by endocytosis, MAP can survive within phagosomes by inhibiting apoptosis, phagosome acidification, and antigen presentation (71). In this context, our findings suggested that the AS of genes involved in endocytosis may contribute to phagosome maturation arrest and MAP persistence within the macrophages of cows with focal lesions in gut tissues.

In the comparison of PB samples from cows with focal lesions *vs.* controls, AS events in genes associated with the pathogenesis of herpesviruses that infect humans at an early age but remain asymptomatic during for long time, such as the Kaposi sarcoma-associated herpesvirus infection (bta05167) and Epstein–Barr virus infection (bta05169), were identified. These genes affected by AS events include the *BOLA*, *LOC509006*, *Mitogen-Activated Protein 2 Kinase 7* (*MAP2K7*), *TNF Receptor Associated Factor 3* (*TRAF3*), *Interferon regulatory factor 7* (*IRF7*), *Phosphatidylinositol-4,5-Bisphosphate 3-Kinase Catalytic Subunit Delta* (*PIK3CD*), and *JSP.1*. IRF7 plays an important role in the regulation of Epstein–Barr virus latency. *TRAF3* can interact with Epstein–Barr virus-encoded latent infection membrane protein-1 (LMP1) along with other members of the TRAF family, acting as a negative NF-kβ regulator and inhibiting the inflammatory response (72). Our findings suggest that the AS of the *BOLA*, *LOC509006*, *MAP2K7*, *TRAF3*, *IRF7*, *PIK3CD*, and *JSP.*1 genes may contribute to the development of focalized PTB-associated lesions and establishment of the latent stage of MAP infection, as it has been observed in Epstein–Barr virus and Kaposi sarcoma-associated herpesvirus latency.

In the comparison of the PB samples from cows with diffuse lesions *vs.* controls, genes involved in the CLTA-mediated endocytosis, neutrophil degranulation, and platelet activation pathways were affected by differential AS. More specifically, AS of genes belonging to the neutrophil degranulation and platelet activation pathways, including the *Cluster of differentiation 53 (CD53), CLEC12A C-type lectin domain family 12 member A* (*CLEC12A*), *Solute Carrier Family 11 Member 1* (*SLC11A1)*, and *T Cell-Interacting, Activating Receptor on Myeloid Cells 1* (*TARM1*) genes was observed. *CD53* contributes to transduction signals in T cells and natural killer cells, and *TARM1* increases the production of pro-inflammatory cytokines in macrophages and neutrophils *via* the toll-like receptor. Neutrophils phagocytose *Mycobacteria* from dying infected macrophages within the granuloma (73, 74). *CLEC12A* and *SLC11A1* are both associated with innate host resistance. SLC11A1 is a membrane transporter involved in iron metabolism expressed in the late endosomal/lysosomal compartments of macrophages. We hypothesize that the AS of genes involved in neutrophil degranulation and platelet activation pathways may, at least in part, contribute to granuloma formation and pro-inflammatory immune response in MAP-infected cattle with diffuse lesions.

In PB samples from cows with diffuse lesions *vs.* focal lesions, several genes (*ACTN1, TGFβ2, ITGβ2*, and *C8G*) with AS events associated with the lysosome (bta:04142) and amoebiasis (bta:05146) were identified. Amoebiasis is caused by *Entamoeba histolytica*, a human extracellular protozoan parasite that, like MAP, invades the intestinal epithelium and over time disrupts the intestinal mucus layer, followed by apoptosis of host epithelial cells (75). MAP infection also impacts the intestinal mucosa and, over time, causes serious damage to the ileum and jejunum, diarrhea, progressive wasting, and the eventual death of the infected animal in the more advanced stages of clinical PTB (76). The AS of several genes associated with the activation of the lysosomes and amoebiasis may also cause disruption of the intestinal mucus layer, apoptosis, and MAP dissemination to other organs and tissues, as seen in humans infected with *Entamoeba histolytica.*

In the ICV samples from the infected cows, genes that encode proteins with RNA-binding domains and coiled-coil domains and those involved in AS showed changes in AS. As RNA splicing is regulated by cis-regulatory elements in pre-mRNA and trans-regulatory elements, mainly RNA-binding proteins, AS of RNA-binding proteins may in turn affect the splicing of other pre-mRNAs and contribute to the various onsets of PTB.

## 5 Associations between miRNAs and AS profiles and gene expression regulation

**Figure 3:**
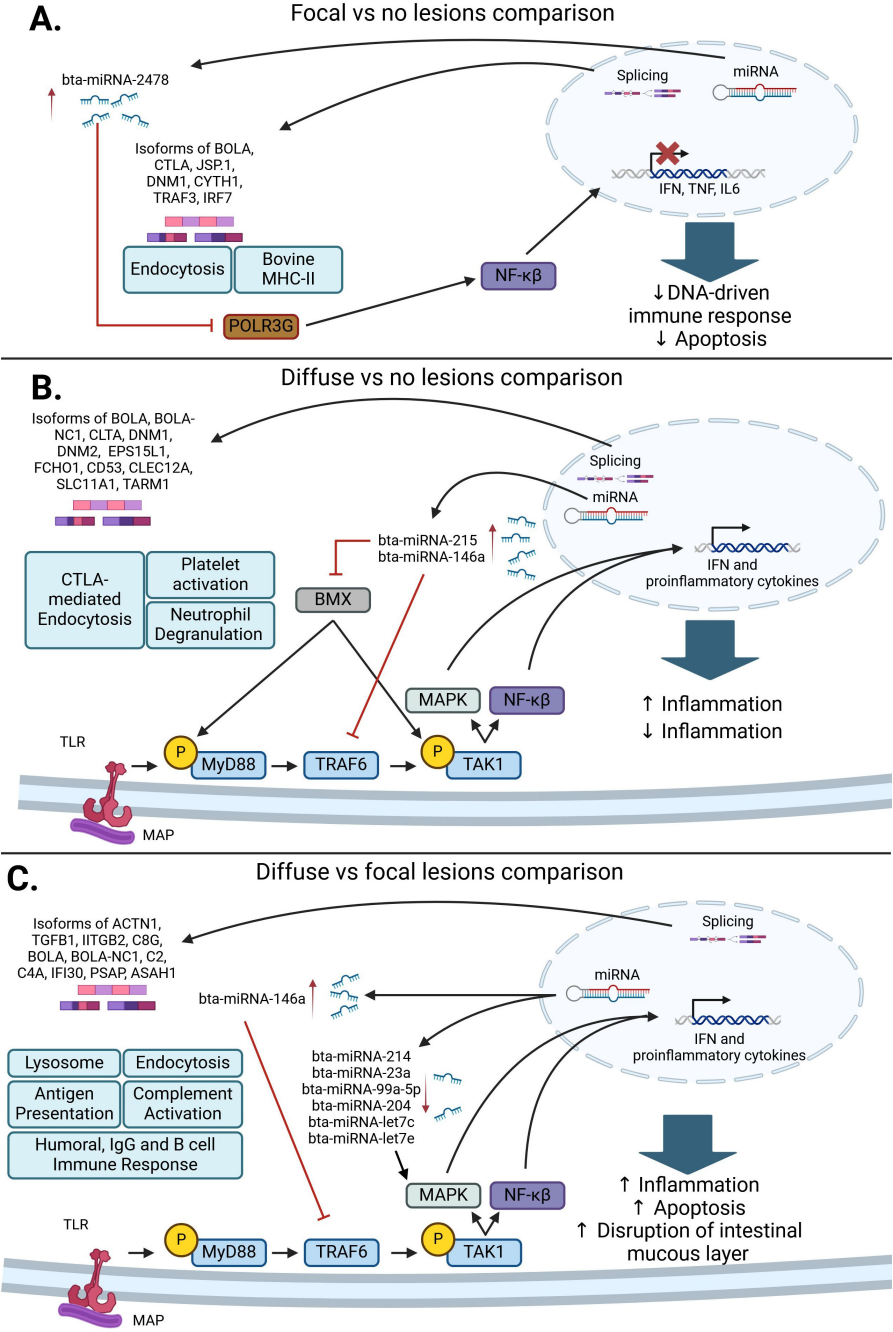
Post-transcriptional regulatory pathways associated with a response to MAP infection. **(A)** In the comparison of cows with focal lesions *vs.* control cows, bta-miRNA-2478 and AS in genes associated with endocytosis and antigen presentation and processing can interfere with NF-κβ and type-I IFN signaling, which would promote MAP survival. **(B)** In the comparison of cows with diffuse *vs.* no lesions, the differentially expressed miRNAs bta-miRNA-215 and bta-miRNA146a inhibit BMX and TRAF6, respectively. The inhibition of these genes causes an inhibition of the MAPK and NF-κβ pathways and a decrease in the production of proinflammatory cytokines. Subsequently, the AS of genes affecting CTLA-mediated endocytosis, platelet activation, and neutrophil degranulation contributes to granuloma formation and the pro-inflammatory response observed in MAP-infected cattle with diffuse lesions. **(C)** In the comparison of cows with diffuse *vs.* focal lesions, a decrease in bta-miRNA-214, bta-miRNA-23a, bta-miRNA-99a-5p, bta-miRNA-204, bta-miRNA-let7c, and bta-miRNA-let7e expression, which target different components of the MAPK pathway, causes an increase in pro-inflammatory cytokine expression. AS events affecting genes associated with endocytosis, the complement system, antigen presentation, the humoral immune response, and the lysosome contribute to an excessive stimulation of the pro-inflammatory response and disruption of the intestinal mucus layer, which results in the development of severe lesions in gut tissues (56, 70) (Created in Biorender.com).

Our previous studies advanced the understanding of gene expression regulation in response to MAP infection, specifically highlighting the roles of miRNAs and AS events (56, 70). Given that both miRNAs and AS contribute to the modulation of gene expression, we integrated the analysis of AS-regulated pathways with the regulatory influence of miRNAs to provide a comprehensive view of the post-transcriptional regulation of gene expression in MAP-infected cattle. In **Figure 3**, a graphical summary of the alternatively spliced genes, miRNAs, and key molecular mechanisms identified is presented. In cows with focal lesions *vs.* control cows (**Figure 3A**), which could be interpreted as the initial stage of MAP infection, both AS events and miRNAs contributed to the inhibition of the innate immune response, apoptosis, and antibacterial response. The response against MAP DNA mediated by Type I IFN, IL-6, and TNF was inhibited by the upregulation of bta-miRNA-2478, which inhibits RNA polymerase III G (*POLR3G*) expression. POLR3G downregulation may be responsible for the blockade of the NF-κβ-mediated innate immune responses that allow for MAP persistence within infected macrophages. The innate immune response was also inhibited via the AS of genes associated with endocytosis, such as *CLTA, CYTH1, JSP.1, RAB11FIP15, DNM2, AP2M1, PRKC1*, and *ARAP1*, and antigen presentation and recognition, such as *BOLA* and *LOC50906*.

In the comparison of cows with diffuse lesions *vs.* control cows (**Figure 3B**), which could be interpreted as the early clinical stage of the infection, upregulation of bta-miRNA-215 and bta-miRNA-146a inhibits the pro-inflammatory immune response via inhibition of BMX and TRAF6, respectively, which allows MAP to persist and grow inside granulomas. In this comparison, AS-affected genes associated with neutrophil degranulation and platelet activation, such as *CD53, CLEC12A, SLC11A1*, and *TARM1*, may stimulate the development of granulomas and the induction of a pro-inflammatory immune response. In the comparison of cows with diffuse lesions *vs.* focal lesions (**Figure 3C**), which could be interpreted as the advanced clinical stage of the infection, the downregulation of bta-miRNA-214, bta-miRNA-23a, bta-miRNA-99a-5p, bta-miRNA-204, bta-miRNA-let7c, and bta-miRNA-let7e promotes the expression of type I IFN and proinflammatory cytokines, causing the activation of a strong pro-inflammatory response. In the same way, AS events differentially expressed in this comparison were associated with endocytosis, antigen presentation, humoral IgG and B cell immune response, and complement activation. Aberrant inflammation and induction of humoral immune response are usually observed in animals with advanced clinical infection. In addition, the AS of genes involved in the lysis of MAP-containing phagosomes was observed in this comparison, which may lead to apoptosis and disruption of the intestinal mucus layer.

## 6 Challenges in RNA-Seq and future directions

RNA-Seq has become a very useful tool for transcriptome profiling. This technology holds significant potential, as it allows for in-depth studies of disease pathogenesis and can be used to identify biomarkers for the development of novel diagnostic tools, drugs, and vaccines. We previously identified host mRNAs, miRNAs, and AS events that were DE in samples from MAP-infected animals with distinct PTB-associated lesions in gut tissues *vs.* controls (26, 56, 70). However, several challenges remain in data interpretation. Technical difficulties arising from RNA library preparation and sequencing depth can introduce data variability, especially in studies with small sample sizes or high biological variability. Accurately quantifying low-abundance transcripts, distinguishing closely related isoforms, and detecting AS events require high sequencing depth and robust bioinformatic pipelines, which are often limited by annotation quality and computational resources.

Looking ahead, future directions in RNA-Seq include the integration of long-read sequencing technologies (e.g., PacBio and Oxford Nanopore) to capture full-length transcripts. Additionally, multi-omics approaches that combine RNA-Seq with genomics (77), proteomics, epigenomics, and spatial transcriptomics have the potential to generate a more holistic understanding of gene regulation. Continued improvement in data integration tools, machine learning-based analysis, and publicly available annotation databases will be critical to fully realizing the potential of RNA-Seq in systems biology and host-pathogen interactions.

Besides the utility of miRNAs and AS events as biomarkers for the detection of subclinical MAP infection, modifying or targeting the expression of the identified miRNAs or AS events may open novel opportunities for the development of miRNAs and/or splicing-based PTB therapies. Novel splicing and miRNA-based therapies are being developed for human inflammatory and autoimmune diseases, but they are still rare in the veterinary context.

## 7 Conclusions

The current diagnostic tools for the direct detection of MAP are fecal real-time qPCR and bacteriological culture. However, both show limitations in detecting subclinical MAP-infected cattle. This review article highlights the contribution of RNA-Seq-based approaches to the discovery of biomarkers that may support both the early diagnosis of MAP infection and an understanding of the regulatory mechanisms involved in the host immune response. It provides a comprehensive review of RNA-Seq studies that explored changes in miRNA expression and AS events in cattle infected with MAP, highlighting key findings in gene expression regulation during MAP infection and offering mechanistic insights into host immune regulation, which will be valuable for researchers in comparative immunology and veterinary science. The review also aimed to compile recent evidence indicating the importance of these regulatory elements in the pathogenesis of PTB, promoting an integrated view of transcriptomic data and its impact on the biology of the bovine immune system. MiRNAs and AS can modulate host responses against MAP infection, allowing MAP survival during the early stage of the infection and promoting granuloma formation, tissue damage, and humoral immune responses in the clinical stages of MAP infection.

## References

[1] World Organization of Animal Health. Terrestrial manual 3.1.15. Paris: OIE (2021). doi: 10.20506/bull.2021.2.3276

[2] Barkema HW Orsel K Nielsen SS Koets AP Rutten VPMG Bannantine JP. Knowledge gaps that hamper prevention and control of *Mycobacterium avium* subspecies *paratuberculosis* infection. Transbound Emerg Dis. (2018) 65:125–48. doi: 10.1111/tbed.12723 28941207

[3] Rasmussen P Barkema HW Mason S Beaulieu E Hall DC. Economic losses due to Johne’s disease (paratuberculosis) in dairy cattle. J Dairy Sci. (2021) 104:3123–43. doi: 10.3168/jds.2020-19381 33455766

[4] Rasmussen P Barkema HW Osei PP Taylor J Shaw AP Conrady B. Global losses due to dairy cattle diseases: A comorbidity-adjusted economic analysis. J Dairy Sci. (2024) 107(9):6945–70. doi: 10.3168/jds.2023-24626 PMC11382338 38788837

[5] Thomas Kennedy Dalziel 1861-1924. Chronic interstitial enteritis. Dis Colon Rectum. (1989) 32(12):1076–8. doi: 10.1007/BF02553886 2686949

[6] Sechi LA Dow CT. Mycobacterium avium ss. paratuberculosis Zoonosis - The Hundred Year War - Beyond Crohn’s Disease. Front Immunol. (2015) 6:96. doi: 10.3389/fimmu.2015.00096 25788897 PMC4349160

[7] Timms VJ Daskalopoulos G Mitchell HM Neilan BA. The Association of Mycobacterium avium subsp. paratuberculosis with Inflammatory Bowel Disease. PloS One. (2016) 11:e0148731. doi: 10.1371/journal.pone.0148731 26849125 PMC4746060

[8] Sechi LA Mura M Tanda E Lissia A Fadda G Zanetti S. Mycobacterium avium sub. paratuberculosis in tissue samples of Crohn’s disease patients. New Microbiol. (2004) 27:75–7. 14964409

[9] Behr MA Kapur V. The evidence for Mycobacterium paratuberculosis in Crohn’s disease. Curr Opin Gastroenterol. (2008) 24:17–21. doi: 10.1097/MOG.0b013e3282f1dcc4 18043227

[10] Juste RA Elguezabal N Garrido JM Pavon A Geijo MV Sevilla I. On the prevalence of M. avium subspecies paratuberculosis DNA in the blood of healthy individuals and patients with inflammatory bowel disease. PloS One. (2008) 3:3–8. doi: 10.1371/journal.pone.0002537 PMC2434204 18596984

[11] Naser S. Isolation of Mycobacterium avium subsp paratuberculosis from breast milk of Crohn’s disease patients. Am J Gastroenterol. (2000) 95:1094–5. doi: 10.1016/S0002-9270(00)00746-2 10763975

[12] Agrawal G Hamblin H Clancy A Borody T. Anti-mycobacterial antibiotic therapy induces remission in active paediatric crohn’s disease. Microorganisms. (2020) 8:1112. doi: 10.3390/microorganisms8081112 32722117 PMC7464505

[13] Mintz MJ Lukin DJ. Mycobacterium avium subspecies paratuberculosis (MAP) and Crohn’s disease: the debate continues. Transl Gastroenterol Hepatol. (2023) 8:28–8. doi: 10.21037/tgh-23-16 PMC10432229 37601744

[14] Naser SA Thanigachalam S Dow C Collins MT. Exploring the role of Mycobacterium avium subspecies paratuberculosis in the pathogenesis of type 1 diabetes mellitus: a pilot study. Gut Pathog. (2013) 5:14. doi: 10.1186/1757-4749-5-14 23759115 PMC3686596

[15] Celler BG. Case Study: Cardiac sarcoidosis resolved with Mycobacterium avium paratuberculosis antibiotics (MAP). Sarcoidosis Vasculitis Diffuse Lung Dis. (2018) 35:171–7. doi: 10.36141/svdld.v35i2.6769 PMC7170090 32476899

[16] Dow CT. Warm, Sweetened Milk at the Twilight of Immunity - Alzheimer’s Disease - Inflammaging, Insulin Resistance, M. paratuberculosis and Immunosenescence. Front Immunol. (2021) 12:714179. doi: 10.3389/fimmu.2021.714179 34421917 PMC8375433

[17] Bo M Jasemi S Uras G Erre GL Passiu G Sechi LA. Role of infections in the pathogenesis of rheumatoid arthritis: Focus on mycobacteria. Microorganisms. (2020) 8:1–19. doi: 10.3390/microorganisms8101459 PMC7598258 32977590

[18] Sisto M Cucci L D’Amore M Dow TC Mitolo V Lisi S. Proposing a relationship between Mycobacterium avium subspecies paratuberculosis infection and Hashimoto’s thyroiditis. Scand J Infect Dis. (2010) 42:787–90. doi: 10.3109/00365541003762306 20429717

[19] Dow CT Ellingson JLE. Detection of Mycobacterium avium ss. Paratuberculosis in Blau Syndrome Tissues. Autoimmune Dis. (2010) 2010:1–5. doi: 10.4061/2010/127692 PMC2989750 21152214

[20] Frau J Cossu D Coghe G Lorefice L Fenu G Melis M. Mycobacterium avium subsp. paratuberculosis and multiple sclerosis in Sardinian patients: epidemiology and clinical features. Multiple Sclerosis J. (2013) 19:1437–42. doi: 10.1177/1352458513477926 23439580

[21] Pierce ES Jindal C Choi YM Cassidy K Efird JT. Pathogenic mechanisms and etiologic aspects of *Mycobacterium avium* subspecies *paratuberculosis* as an infectious cause of cutaneous melanoma. MedComm – Oncol. (2024) 3(2):e72. doi: 10.1002/mog2.72 38831791 PMC11145504

[22] Pierce ES Jindal C Choi YM Efird JT. The evidence for Mycobacterium avium subspecies paratuberculosis (MAP) as a cause of nonsolar uveal melanoma: a narrative review. Transl Cancer Res. (2023) 12:398–412. doi: 10.21037/tcr-22-2540 36915598 PMC10007888

[23] Pierce ES. Could Mycobacterium avium subspecies paratuberculosis cause Crohn’s disease, ulcerative colitis … and colorectal cancer? Infect Agent Cancer. (2018) 13:1–6. doi: 10.1186/s13027-017-0172-3 29308085 PMC5753485

[24] Hempel RJ Bannantine JP Stabel JR. Transcriptional Profiling of Ileocecal Valve of Holstein Dairy Cows Infected with Mycobacterium avium subsp. Paratuberculosis. PloS One. (2016) 11:e0153932. doi: 10.1371/journal.pone.0153932 27093613 PMC4836751

[25] Liang G Malmuthuge N Guan Y Ren Y Griebel PJ Guan LL. Altered microRNA expression and pre-mRNA splicing events reveal new mechanisms associated with early stage Mycobacterium avium subspecies paratuberculosis infection. Sci Rep. (2016) 6:24964. doi: 10.1038/srep24964 27102525 PMC4840452

[26] Alonso-Hearn M Canive M Blanco-Vazquez C Torremocha R Balseiro A Amado J. RNA-Seq analysis of ileocecal valve and peripheral blood from Holstein cattle infected with Mycobacterium avium subsp. paratuberculosis revealed dysregulation of the CXCL8/IL8 signaling pathway. Sci Rep. (2019) 9:14845. doi: 10.1038/s41598-019-51328-0 31619718 PMC6795908

[27] Mallikarjunappa S Adnane M Cormican P Karrow NA Meade KG. Characterization of the bovine salivary gland transcriptome associated with Mycobacterium avium subsp. paratuberculosis experimental challenge. BMC Genomics. (2019) 20:491. doi: 10.1186/s12864-019-5845-4 31195975 PMC6567491

[28] Malvisi M Curti N Remondini D De Iorio MG Palazzo F Gandini G. Combinatorial Discriminant Analysis Applied to RNAseq Data Reveals a Set of 10 Transcripts as Signatures of Exposure of Cattle to Mycobacterium avium subsp. paratuberculosis. Animals. (2020) 10:253. doi: 10.3390/ani10020253 32033399 PMC7070263

[29] Facciuolo A Lee AH Gonzalez Cano P Townsend HGG Falsafi R Gerdts V. Regional Dichotomy in Enteric Mucosal Immune Responses to a Persistent Mycobacterium avium ssp. paratuberculosis Infection. Front Immunol. (2020) 11:1020. doi: 10.3389/fimmu.2020.01020 32547548 PMC7272674

[30] Ibeagha-Awemu EM Bissonnette N Do DN Dudemaine P-L Wang M Facciuolo A. Regionally Distinct Immune and Metabolic Transcriptional Responses in the Bovine Small Intestine and Draining Lymph Nodes During a Subclinical Mycobacterium avium subsp. paratuberculosis Infection. Front Immunol. (2021) 12:760931. doi: 10.3389/fimmu.2021.760931 34975852 PMC8714790

[31] Casey ME Meade KG Nalpas NC Taraktsoglou M Browne JA Killick KE. Analysis of the bovine monocyte-derived macrophage response to mycobacterium avium subspecies paratuberculosis infection using RNA-seq. Front Immunol. (2015) 6:23. doi: 10.3389/fimmu.2015.00023 25699042 PMC4316787

[32] Marino R Capoferri R Panelli S Minozzi G Strozzi F Trevisi E. Johne’s disease in cattle: an *in vitro* model to study early response to infection of Mycobacterium avium subsp. paratuberculosis using RNA-seq. Mol Immunol. (2017) 91:259–71. doi: 10.1016/j.molimm.2017.08.017 28988040

[33] Gupta P Peter S Jung M Lewin A Hemmrich-Stanisak G Franke A. Analysis of long non-coding RNA and mRNA expression in bovine macrophages brings up novel aspects of *Mycobacterium avium* subspecies *paratuberculosis* infections. Sci Rep. (2019) 9:1571. doi: 10.1038/s41598-018-38141-x 30733564 PMC6367368

[34] Ariel O Gendron D Dudemaine P-L Gévry N Ibeagha-Awemu EM Bissonnette N. Transcriptome Profiling of Bovine Macrophages Infected by Mycobacterium avium spp. paratuberculosis Depicts Foam Cell and Innate Immune Tolerance Phenotypes. Front Immunol. (2020) 10:2874. doi: 10.3389/fimmu.2019.02874 31969876 PMC6960179

[35] Ariel O Brouard J-S Marete A Miglior F Ibeagha-Awemu E Bissonnette N. Genome-wide association analysis identified both RNA-seq and DNA variants associated to paratuberculosis in Canadian Holstein cattle ‘*in vitro*’ experimentally infected macrophages. BMC Genomics. (2021) 22:162. doi: 10.1186/s12864-021-07487-4 33678157 PMC7938594

[36] Bao Y Wu S Yang T Wang Z Wang Y Jiang X. Analysis of long non-coding RNA expression profile of bovine monocyte-macrophage infected by *Mycobacterium avium* subsp. *paratuberculosis* . BMC Genomics. (2022) 23(1):768. doi: 10.1186/s12864-022-08997-5 36418939 PMC9685057

[37] Park H-T Park WB Kim S Lim J-S Nah G Yoo HS. Revealing immune responses in the Mycobacterium avium subsp. paratuberculosis-infected THP-1 cells using single cell RNA-sequencing. PloS One. (2021) 16:e0254194. doi: 10.1371/journal.pone.0254194 34214113 PMC8253428

[38] Park H-E Lee W Choi S Jung M Shin M-K Shin SJ. Modulating macrophage function to reinforce host innate resistance against Mycobacterium avium complex infection. Front Immunol. (2022) 13:931876. doi: 10.3389/fimmu.2022.931876 36505429 PMC9730288

[39] Blanco-Vázquez C Alonso-Hearn M Iglesias N Vázquez P Juste RA Garrido JM. Use of ATP-binding cassette subfamily A member 13 (ABCA13) for sensitive detection of focal pathological forms of subclinical bovine paratuberculosis. Front Vet Sci. (2022) 9:816135. doi: 10.3389/fvets.2022.816135 35359676 PMC8960928

[40] Lee RC Feinbaum RL Ambros V. The C. elegans heterochronic gene lin-4 encodes small RNAs with antisense complementarity to lin-14. Cell. (1993) 75:843–54. doi: 10.1016/0092-8674(93)90529-Y 8252621

[41] Miyoshi K Miyoshi T Siomi H. Many ways to generate microRNA-like small RNAs: non-canonical pathways for microRNA production. Mol Genet Genomics. (2010) 284:95–103. doi: 10.1007/s00438-010-0556-1 20596726

[42] Kim VN. MicroRNA biogenesis: coordinated cropping and dicing. Nat Rev Mol Cell Biol. (2005) 6:376–85. doi: 10.1038/nrm1644 15852042

[43] Han J Lee Y Yeom K-H Kim Y-K Jin H Kim VN. The Drosha-DGCR8 complex in primary microRNA processing. Genes Dev. (2004) 18:3016–27. doi: 10.1101/gad.1262504 PMC535913 15574589

[44] Peng Y Croce CM. The role of MicroRNAs in human cancer. Signal Transduct Target Ther. (2016) 1:15004. doi: 10.1038/sigtrans.2015.4 29263891 PMC5661652

[45] O’Brien J Hayder H Zayed Y Peng C. Overview of microRNA biogenesis, mechanisms of actions, and circulation. Front Endocrinol (Lausanne). (2018) 9:402. doi: 10.3389/fendo.2018.00402 30123182 PMC6085463

[46] Fang Z Rajewsky N. The impact of miRNA target sites in coding sequences and in 3′UTRs. PloS One. (2011) 6(3):e18067. doi: 10.1371/journal.pone.0018067 21445367 PMC3062573

[47] Turchinovich A Weiz L Burwinkel B. Extracellular miRNAs: The mystery of their origin and function. Trends Biochem Sci. (2012) 37:460–5. doi: 10.1016/j.tibs.2012.08.003 22944280

[48] Farrell D Shaughnessy RG Britton L MacHugh DE Markey B Gordon SV. The identification of circulating MiRNA in bovine serum and their potential as novel biomarkers of early mycobacterium avium subsp paratuberculosis infection. PloS One. (2015) 10:1–22. doi: 10.1371/journal.pone.0134310 PMC4517789 26218736

[49] Wang Z Kong LC Jia BY Chen JR Dong Y Jiang XY. Analysis of the microRNA Expression Profile of Bovine Monocyte-derived Macrophages Infected with Mycobacterium avium subsp. Paratuberculosis Reveals that miR-150 Suppresses Cell Apoptosis by Targeting PDCD4. Int J Mol Sci. (2019) 20:2708. doi: 10.3390/ijms20112708 31159463 PMC6600136

[50] Wright K Mizzi R Plain KM Purdie AC de Silva K. Mycobacterium avium subsp. paratuberculosis exploits miRNA expression to modulate lipid metabolism and macrophage polarisation pathways during infection. Sci Rep. (2022) 12:9681. doi: 10.1038/s41598-022-13503-8 35690602 PMC9188571

[51] Shandilya UK Wu X McAllister C Mutharia L Karrow NA. Impact of Mycobacterium avium subsp. paratuberculosis infection on bovine IL10RA knockout mammary epithelial (MAC-T) cells. In Vitro Cell Dev Biol Anim. (2023) 59:214–23. doi: 10.1007/s11626-023-00758-2 37071310

[52] Malvisi M Palazzo F Morandi N Lazzari B Williams JL Pagnacco G. Responses of bovine innate immunity to mycobacterium avium subsp. Paratuberculosis infection revealed by changes in gene expression and levels of MicroRNA. PloS One. (2016) 11:1–23. doi: 10.1371/journal.pone.0164461 PMC5070780 27760169

[53] Shaughnessy RG Farrell D Stojkovic B Browne JA Kenny K Gordon SV. Identification of microRNAs in bovine faeces and their potential as biomarkers of Johne’s Disease. Sci Rep. (2020) 10:5908. doi: 10.1038/s41598-020-62843-w 32246047 PMC7125074

[54] Choi SW Kim S Park HT Park HE Choi JS Yoo HS. MicroRNA profiling in bovine serum according to the stage of *Mycobacterium avium* subsp. *paratuberculosis* infection. PloS One. (2021) 16(11):e0259539. doi: 10.1371/journal.pone.0259539 34735546 PMC8568169

[55] Gupta SK Maclean PH Ganesh S Shu D Buddle BM Wedlock DN. Detection of microRNA in cattle serum and their potential use to diagnose severity of Johne’s disease. J Dairy Sci. (2018) 101:10259–70. doi: 10.3168/jds.2018-14785 30197143

[56] Badia-Bringué G Canive M Blanco-Vázquez C Torremocha R Ovalle S Ramos-Ruiz R. MicroRNAs modulate immunological and inflammatory responses in Holstein cattle naturally infected with Mycobacterium avium subsp. paratuberculosis. Sci Rep. (2024) 14:173. doi: 10.1038/s41598-023-50251-9 38167436 PMC10762146

[57] Chandan K Gupta M Sarwat M. Role of host and pathogen-derived microRNAs in immune regulation during infectious and inflammatory diseases. Front Immunol. (2020) 10:3081. doi: 10.3389/fimmu.2019.03081 32038627 PMC6992578

[58] Quinn EM Wang JH O’Callaghan G Redmond HP. MicroRNA-146a is upregulated by and negatively regulates TLR2 signaling. PloS One. (2013) 8(4):e62232. doi: 10.1371/journal.pone.0062232 23638011 PMC3639252

[59] Furci L Schena E Miotto P Cirillo DM. Alteration of human macrophages microRNA expression profile upon infection with *Mycobacterium tuberculosis* . Int J Mycobacteriol. (2013) 2:128–34. doi: 10.1016/j.ijmyco.2013.04.006 26785980

[60] Vegh P Magee DA Nalpas NC Bryan K McCabe MS Browne JA. MicroRNA profiling of the bovine alveolar macrophage response to *Mycobacterium bovis* infection suggests pathogen survival is enhanced by microRNA regulation of endocytosis and lysosome trafficking. Tuberculosis. (2015) 95:60–7. doi: 10.1016/j.tube.2014.10.011 25692199

[61] Wahl MC Will CL Lührmann R. The spliceosome: design principles of a dynamic RNP machine. Cell. (2009) 136:701–18. doi: 10.1016/j.cell.2009.02.009 19239890

[62] Lee Y Rio DC. Mechanisms and regulation of alternative pre-mRNA splicing. Annu Rev Biochem. (2015) 84:291–323. doi: 10.1146/annurev-biochem-060614-034316 25784052 PMC4526142

[63] Dvinge H Kim E Abdel-Wahab O Bradley RK. RNA splicing factors as oncoproteins and tumour suppressors. Nat Rev Cancer. (2016) 16:413–30. doi: 10.1038/nrc.2016.51 PMC5094465 27282250

[64] Rotival M Quach H Quintana-Murci L. Defining the genetic and evolutionary architecture of alternative splicing in response to infection. Nat Commun. (2019) 10:1671. doi: 10.1038/s41467-019-09689-7 30975994 PMC6459842

[65] Tabrez SS Sharma RD Jain V Siddiqui AA Mukhopadhyay A. Differential alternative splicing coupled to nonsense-mediated decay of mRNA ensures dietary restriction-induced longevity. Nat Commun. (2017) 8:306. doi: 10.1038/s41467-017-00370-5 28824175 PMC5563511

[66] Wang G-S Cooper TA. Splicing in disease: disruption of the splicing code and the decoding machinery. Nat Rev Genet. (2007) 8:749–61. doi: 10.1038/nrg2164 17726481

[67] Zhong XZ Zou Y Sun X Dong G Cao Q Pandey A. Inhibition of transient receptor potential channel mucolipin-1 (TRPML1) by lysosomal adenosine involved in severe combined immunodeficiency diseases. J Biol Chem. (2017) 292:3445–55. doi: 10.1074/jbc.M116.743963 PMC5336176 28087698

[68] Liu Q Zheng J Yin D-D Xiang J He F Wang Y-C. Monocyte to macrophage differentiation-associated (MMD) positively regulates ERK and Akt activation and TNF-α and NO production in macrophages. Mol Biol Rep. (2012) 39:5643–50. doi: 10.1007/s11033-011-1370-5 22203480

[69] Li H Huang J Zhang J Gao Y Han B Sun D. Identification of alternative splicing events associated with paratuberculosis in dairy cattle using multi-tissue RNA sequencing data. Genes (Basel). (2022) 13:497. doi: 10.3390/genes13030497 35328051 PMC8948961

[70] Badia-Bringué G Lavín JL Casais R Alonso-Hearn M. Alternative splicing of pre-mRNA modulates the immune response in Holstein cattle naturally infected with Mycobacterium avium subsp. paratuberculosis. Front Immunol. (2024) 15:1354500. doi: 10.3389/fimmu.2024.1354500 38495873 PMC10940349

[71] Khare S Lawhon SD Drake KL Nunes JES Figueiredo JF Rossetti CA. Systems Biology Analysis of Gene Expression during *In Vivo* Mycobacterium avium paratuberculosis Enteric Colonization Reveals Role for Immune Tolerance. PloS One. (2012) 7:e42127. doi: 10.1371/journal.pone.0042127 22912686 PMC3422314

[72] Ning S Pagano JS Barber GN. IRF7: activation, regulation, modification and function. Genes Immun. (2011) 12:399–414. doi: 10.1038/gene.2011.21 21490621 PMC4437765

[73] Yang CT Cambier CJ Davis JM Hall CJ Crosier PS Ramakrishnan L. Neutrophils exert protection in the early tuberculous granuloma by oxidative killing of mycobacteria phagocytosed from infected macrophages. Cell Host Microbe. (2012) 12:301–12. doi: 10.1016/j.chom.2012.07.009 PMC3638950 22980327

[74] Futosi K Fodor S Mócsai A. Neutrophil cell surface receptors and their intracellular signal transduction pathways. Int Immunopharmacol. (2013) 17:638–50. doi: 10.1016/j.intimp.2013.06.034 PMC3827506 23994464

[75] Sellau J Groneberg M Hoenow S Lotter H. The underlying cellular immune pathology of Entamoeba histolytica-induced hepatic amoebiasis. J Hepatol. (2021) 75:481–2. doi: 10.1016/j.jhep.2021.03.018 34120776

[76] Sweeney RW. Pathogenesis of paratuberculosis. Veterinary Clinics North America: Food Anim Pract. (2011) 27:537–46. doi: 10.1016/j.cvfa.2011.07.001 22023832

[77] Badia-Bringué G Canive M Fernandez-Jimenez N Lavín JL Casais R Blanco-Vázquez C. Summary-data based Mendelian randomization identifies gene expression regulatory polymorphisms associated with bovine paratuberculosis by modulation of the nuclear factor Kappa β (NF-κß)-mediated inflammatory response. BMC Genomics. (2023) 24(1):605. doi: 10.1186/s12864-023-09710-w 37821814 PMC10568764

